# *BrainAGE* in Mild Cognitive Impaired Patients: Predicting the Conversion to Alzheimer’s Disease

**DOI:** 10.1371/journal.pone.0067346

**Published:** 2013-06-27

**Authors:** Christian Gaser, Katja Franke, Stefan Klöppel, Nikolaos Koutsouleris, Heinrich Sauer, Alzheimer's Disease Neuroimaging Initiative

**Affiliations:** 1 Structural Brain Mapping Group, Department of Psychiatry, Jena University Hospital, Jena, Germany; 2 Department of Neurology, Jena University Hospital, Jena, Germany; 3 Department of Psychiatry and Psychotherapy, Department of Neurology, Freiburg Brain Imaging, University Medical Center Freiburg, Freiburg, Germany; 4 Department of Psychiatry and Psychotherapy, Ludwig-Maximilians-University, Munich, Germany; Nathan Kline Institute and New York University School of Medicine, United States of America

## Abstract

Alzheimer’s disease (AD), the most common form of dementia, shares many aspects of abnormal brain aging. We present a novel magnetic resonance imaging (MRI)-based biomarker that predicts the individual progression of mild cognitive impairment (MCI) to AD on the basis of pathological brain aging patterns. By employing kernel regression methods, the expression of normal brain-aging patterns forms the basis to estimate the brain age of a given new subject. If the estimated age is higher than the chronological age, a positive brain age gap estimation (BrainAGE) score indicates accelerated atrophy and is considered a risk factor for conversion to AD. Here, the BrainAGE framework was applied to predict the individual brain ages of 195 subjects with MCI at baseline, of which a total of 133 developed AD during 36 months of follow-up (corresponding to a pre-test probability of 68%). The ability of the BrainAGE framework to correctly identify MCI-converters was compared with the performance of commonly used cognitive scales, hippocampus volume, and state-of-the-art biomarkers derived from cerebrospinal fluid (CSF). With accuracy rates of up to 81%, BrainAGE outperformed all cognitive scales and CSF biomarkers in predicting conversion of MCI to AD within 3 years of follow-up. Each additional year in the BrainAGE score was associated with a 10% greater risk of developing AD (hazard rate: 1.10 [CI: 1.07–1.13]). Furthermore, the post-test probability was increased to 90% when using baseline BrainAGE scores to predict conversion to AD. The presented framework allows an accurate prediction even with multicenter data. Its fast and fully automated nature facilitates the integration into the clinical workflow. It can be exploited as a tool for screening as well as for monitoring treatment options.

## Background

The global prevalence of dementia is projected to rise sharply over the coming decades. By 2050, 1 in 85 persons worldwide will be affected by Alzheimer’s disease (AD), the most common form of dementia [1]. Manifold pathological changes begin to develop years or decades before the onset of cognitive decline [2], including premature changes in gene expression [3], [4], accelerated age-associated changes of the default mode network [5], and most obviously, abnormal changes in brain structures already at the mild cognitive impairment (MCI) stage [6], [7]. Additionally, atrophic regions detected in AD patients were recently found to largely overlap with those regions showing a normal age-related decline in healthy control subjects [8].

Early detection and quantification of abnormal brain changes is important for the prospective identification and subsequent treatment of individuals at risk for cognitive decline and dementia. The best validated biomarkers for an early detection include markers of brain β-amyloid-plaque (Aβ) deposition, i.e. decreased CSF Aβ_42_ and positive Pittsburgh compound B (PiB) amyloid imaging, as well as markers of neurodegeneration, i.e. increased CSF tau, decreased fluorodeoxyglucose uptake on PET (FDG-PET), and structural magnetic resonance imaging (MRI) measures of cerebral atrophy [2]. More specifically, low concentrations of CSF Aβ_42_, associated with the formation of Aβ plaques in the brain, were found to correlate with the clinical diagnosis of AD [9], [10], but not with rates of brain atrophy [11]. The process of Aβ-plaque accumulation begins at least 5–10 years [12] or even up to two decades before probable manifestation of clinical symptoms and conversion to AD [13], but on its own is not sufficient to cause dementia [2], [14]–[18]. At some point in the AD disease course accelerated neurodegeneration takes place, preceding accelerated cognitive decline [2]. Although CSF tau was found to positively correlate with severity of cognitive impairment [12], [19], increased CSF tau is not specific for AD but seems to indicate neuronal injury and neurodegeneration in general [2], [20], [21]. Although brain atrophy in general is not specific for AD, MRI-detected atrophy was found to retain the closest relationship with cognitive decline [2], [22], [23] suggesting a crucial role for structural MRI in predicting future conversion to AD [2], [24].

**Figure 1:**
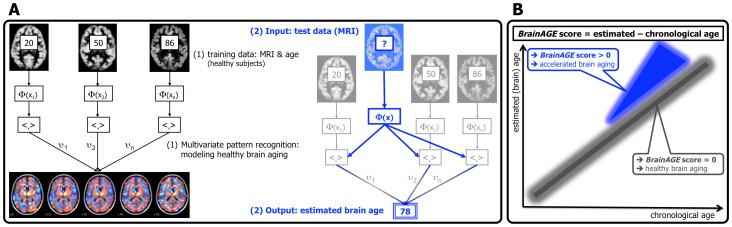
Depiction of the *BrainAGE* concept. (A) The model of healthy brain aging is trained with the chronological age and preprocessed structural MRI data of a training sample (left; with an exemplary illustration of the most important voxel locations that were used by the age regression model). Subsequently, the individual brain ages of previously unseen test subjects are estimated, based on their MRI data (blue; picture modified from Schölkopf & Smola, 2002 [35]). (B) The difference between the estimated and chronological age results in the *BrainAGE* score, indicating abnormal brain aging. [Image reproduced from Franke & Gaser, 2012 [27], with permission from Hogrefe Publishing, Bern].

Our recently introduced *BrainAGE* approach [25], [26] takes into account the widespread but sequential age-related brain tissue loss. Based on single time-point structural MRI the complex, multidimensional aging patterns across the whole brain are aggregated to one single value, i.e. the estimated brain age (Figure 1A). Consequently, although using only a standard MRI scan, the deviation in brain atrophy from normal brain aging can be directly quantified (Fig. 1B). We already demonstrated that the *BrainAGE* approach is capable of identifying pathological brain aging in subjects with MCI and AD, and observed profound relationships between *BrainAGE,* disease severity and prospective worsening of cognitive functions [27].

In order to explore the potential of applying the *BrainAGE* approach in early detection of abnormal brain changes, this study implemented this novel MRI-based biomarker to predict the conversion from MCI to AD within a time span of 36 months. We hypothesized that those individuals with greater *BrainAGE* scores would convert to AD with worse outcomes related to cognition and disease severity. Furthermore, a subsample of subjects with MCI, for whom CSF data are available, will be used to compare the performance of the *BrainAGE* framework in predicting conversion from MCI to AD to commonly used MRI and CSF biomarkers, which are widely used as state-of-the-art benchmark.

## Methods

### Subjects

We utilized data obtained from the ADNI database (www.loni.ucla.edu/ADNI), including all MCI subjects for whom baseline MRI data (1.5T), at least moderately confident diagnoses (i.e. confidence >2), hippocampus volumes (i.e. volumes of left and right hippocampus, calculated by FreeSurfer Version 4.3.), and test scores in certain cognitive scales (i.e. ADAS: Alzheimer’s Disease Assessment Scale, range 0–85; CDR-SB: Clinical Dementia Rating ‘sum of boxes’, range 0–18; MMSE: Mini-Mental State Examination, range 0–30) were available (data downloaded in May 2010). For the exact procedures of data collection and up-to-date information, see www.adni-info.org.

Adopting the diagnostic classification at baseline and follow-up, 195 subjects were grouped as (i) *sMCI* (stable MCI), if diagnosis was MCI at all available time points, but at least for 36 months (n = 62); (ii) *pMCI_early* (progressive MCI), if diagnosis was MCI at baseline but converted to AD within the first 12 months, without reversion to MCI or cognitive normal (NO) at any available follow-up (n = 58); (iii) *pMCI_late*, if diagnosis was MCI at baseline and conversion to AD was reported after the first 12 months (i.e. at 18, 24, or 36 months follow-up), without reversion to MCI or NO at any available follow-up (n = 75). Details of the characteristics of the ADNI test sample are presented in Table 1.

**Table 1:** Baseline characteristics of the MCI samples used in this study.

	Whole sample (n = 195)				CSF subsample (n = 99)				*F* statistic (group x subsample)
	pMCI_early	pMCI_late	sMCI	*F* statistic (group)	pMCI^CSF^_ early	pMCI^CSF^_late	sMCI^CSF^	*F* statistic (group)	
No. subjects	58	75	62	–	32	34	33	–	–
Males/Females	33/25	48/27	49/13	–	18/14	13/11	27/6	–	–
Age range	55–86	56–88	58–88	–	55–86	58–88	63–88	–	–
Age mean	73.9	75.2	76.4	1.85	73.4	76.3	76.3	1.88	0.83
(SD)	(7.0)	(7.3)	(6.2)	[p = 0.16]	(7.0)	(7.7)	(5.8)	[p = 0.16]	[p = 0.44]
Education years mean	15.4	16.0	16.5	2.24	15.2	15.7	16.6	1.84	0.29
(SD)	(2.9)	(2.9)	(2.6)	[p = 0.11]	(3.1)	(3.0)	(2.4)	[p = 0.16]	[p = 0.75]
MMSE mean	26.5	26.8	27.7	**8.67**	26.4	26.6	27.4	2.58	0.46
(SD)	(1.9)	(1.6)	(1.8)	**[p<0.001]**	(2.0)	(1.6)	(1.8)	[p = 0.08]	[p = 0.63]
CDR-SB mean	2.0	1.8	1.3	**8.97**	2.0	1.7	1.3	**4.23**	0.07
(SD)	(0.9)	(1.0)	(0.7)	**[p<0.001]**	(0.9)	(1.1)	(0.6)	**[p<0.05]**	[p = 0.93]
ADAS mean	23.5	20.4	16.3	**26.60**	22.9	20.3	16.5	**10.72**	0.45
(SD)	(6.3)	(4.3)	(5.8)	**[p<0.001]**	(5.7)	(4.4)	(6.3)	**[p<0.001]**	[p = 0.63]
Left hippocampus volume mean	2908.7	2923.4	3260.6	**9.75**	2821.4	2906.9	3273.1	**8.09**	0.76
(SD)	(473.1)	(550.9)	(478.7)	**[p<0.001]**	(482.0)	(516.3)	(446.9)	**[p<0.001]**	[p = 0.47]
Right hippocampus volume mean	2950.4	2963.4	3275.6	**8.07**	2873.5	2877.8	3255.2	**7.07**	0.29
(SD)	(506.1)	(550.7)	(476.0)	**[p<0.001]**	(446.8)	(528.9)	(435.8)	**[p<0.01]**	[p = 0.75]
T-Tau mean	–	–	–	–	113.8	116.8	99.2	1.11	–
(SD)					(54.6)	(46.6)	(54.1)	[p = 0.33]	
P-Tau mean	–	–	–	–	44.7	39.2	34.9	2.77	–
(SD)					(17.1)	(15.6)	(18.0)	[p = 0.07]	
Aβ_42_ mean	–	–	–	–	142.5	147.3	168.5	2.77	–
(SD)					(35.7)	(38.2)	(63.9)	[p = 0.07]	

**Bold** type = significant test results.

To compare the performance of the *BrainAGE* framework in predicting conversion from MCI to AD to the commonly used CSF biomarkers Aβ_42_, total and phosphorylated tau (T-Tau and P-Tau), a subsample of subjects with MCI, for whom those CSF data are available, is utilized (Table 1). Adopting the same criteria as described above, this subsample is grouped as *sMCI^CSF^* (n = 33), *pMCI^CSF^_early* (n = 32), and *pMCI^CSF^_late* (n = 34). In terms of the main baseline characteristics (i.e., age, gender, education, cognition, hippocampus volumes), the CSF subsample was representative of the whole MCI sample used in this study (see Table 1).

To train and test the age estimation framework with respect to prediction accuracy and reliability, we used MRI data of healthy subjects from the publicly accessible IXI cohort (http://www.brain-development.org; data downloaded in February 2009) aged 50 years and older. To evaluate the accuracy of the age estimations, the subjects were divided into training and evaluation samples, i.e. after sorting the subjects by age every fourth subject entered the evaluation sample. Since the number of training samples was found to have the strongest influence on the accuracy of age prediction, MRI data of healthy subjects from the publicly accessible database OASIS (http://www.oasis-brains.org; data downloaded in June 2009) aged 50 years and older were also included in the training sample. In sum the training sample includes 320 cognitive normal elderly subjects. Details of the characteristics of the training sample are presented in Table 2.

**Table 2:** Characteristics of the samples used to model normal brain aging.

	Training sample (n = 320)		Evaluation sample (IXI)
	IXI	OASIS	
No. subjects	194	126	64
Males/Females	72/122	35/91	24/40
Age mean (SD)	63.5 (7.6)	71.3 (11.8)	63.5 (7.5)
Age range	51–86	51–94	51–83

### Preprocessing of MRI Data and Data Reduction

Preprocessing of the T1-weighted images was done using the SPM8 package (http://www.fil.ion.ucl.ac.uk/spm) and the VBM8 toolbox (http://dbm.neuro.uni-jena.de), running under Matlab. All T1-weighted images were corrected for bias-field inhomogeneities, then spatially normalized and segmented into grey matter, white matter, and CSF within the same generative model [28]. The segmentation procedure was further extended by accounting for partial volume effects [29], by applying adaptive maximum a posteriori estimations [30], and by using a hidden Markov random field model [31] as described previously [32]. Only grey matter images were used. Following the pipeline proposed by Franke et al. [25], the images were processed with affine registration and smoothed with 8-mm full-width-at-half-maximum smoothing kernels. After smoothing, spatial resolution was set to 8 mm. Then, data reduction was performed by applying principal component analysis (PCA), utilizing the ‘Matlab Toolbox for Dimensionality Reduction’ (http://ict.ewi.tudelft.nl/~lvandermaaten/Home.html). PCA was only performed on the training sample and the estimated transformation parameters were subsequently applied to the test sample. No further data reduction or region pre-selection was accomplished.

### Relevance Vector Regression (RVR)

Relevance vector machines (RVM) were introduced by Tipping [33] as a Bayesian alternative to support vector machines (SVM) for obtaining sparse solutions to pattern recognition tasks. The main idea behind SVMs is the transformation of training data from input space into high-dimensional space – the *feature space* – via a mapping function Φ [34], [35]. For the purpose of classification, the hyperplane that best separates the groups is computed within this feature space, resulting in a nonlinear decision boundary within the input space. The best separating hyperplane is found by maximizing the margin between the two groups. The data points lying on the margin boundaries are called *support vectors* since only these are used to specify the optimal separating hyperplane. For the case of real-valued output functions (rather than just binary outputs as used in classification), the SV algorithm was generalized to regression estimation [34], [35]. In support vector regression (SVR), a function has to be found that fits as many data points as possible. Analogous to the margin in classification, the regression line is surrounded by a tube. Data points lying within that tube do not influence the course of the regression line. Data points lying on the edge or outside that tube are called *support vectors*.

In contrast to the support vectors in SVM, the *relevance vectors* in RVM represent the prototypical examples within the specified classification or regression task, instead of solely representing separating attributes. Furthermore, severe overfitting associated with the maximum likelihood estimation of the model parameters was avoided by imposing an explicit zero-mean Gaussian prior [36], [37]. This prior is a characteristic feature of the RVM, and its use results in a vector of independent hyperparameters that reduces the data set [33], [38], [39]. Therefore, in most cases the number of relevance vectors is much smaller than the number of support vectors. Furthermore, in SVR additional parameters have to be determined or statistically optimized (e.g. with cross-validation loops) in order to control for model complexity and model fit. To control the behavior of the RVR, only the type of kernel has to be chosen, whereas all other parameters are automatically estimated by the learning procedure itself. More details can be found in [33], [35], [40].

### Age Estimation Framework

The *BrainAGE* framework utilizes RVR [41] and was recently developed to estimate individual brain ages based on T1-weighted images [25]. As suggested by Franke et al. [25], the kernel was chosen to be a polynomial of degree 1, since age estimation accuracy was shown to not improve when choosing non-linear kernels. Thus, parameter optimization during the training procedure was not necessary.

In general, the model is trained with preprocessed whole brain structural MRI data (as described above) of the training sample. Subsequently, the brain age of a test subject can be estimated using the individual tissue-classified MRI data (as described above), aggregating the complex, multidimensional aging pattern across the whole brain into one single value. The difference between estimated and true chronological age will reveal the individual brain age gap estimation (*BrainAGE*) score. Consequently, the *BrainAGE* score directly quantifies the amount of acceleration or deceleration of brain aging. For example, if a 70 years old individual has a *BrainAGE* score of +5 years, this means that this individual shows the typical atrophy pattern of a 75 years old individual. For training the model as well as for predicting individual brain ages, we used “The Spider” (http://www.kyb.mpg.de/bs/people/spider/main.html), a freely available toolbox running under MATLAB. More detailed information as well as the most important features data that were used by the RVR for estimating the brain age can be found in Franke et al. [25].

Within this study, the framework was separately trained on male and female subjects in the training sample. With a mean absolute error of 3.8 years in the evaluation sample of healthy subjects the framework showed accurate performance in brain age estimation. Subsequently, the brain ages of the test subjects were estimated based on their baseline MRI data. The difference between the estimated and the true age resulted in the *BrainAGE* score, indicating accelerated (positive values) or decelerated (negative values) brain aging. PCA was performed on the training sample and the estimated transformation parameters were subsequently applied to the test subjects.

### Statistical Analysis

The baseline *BrainAGE* scores as well as the cognitive scores (i.e. MMSE, CDR-SB, ADAS), the hippocampus volumes, and the CSF biomarker levels at baseline were compared between the diagnostic groups in both MCI test samples using an analysis of variance (ANOVA). To assess the relationship between *BrainAGE* and cognitive measures at baseline and follow-up, Pearson’s pairwise correlation was computed.

Receiver operating characteristics (ROC) for discriminating MCI subjects who converted to AD from those who remained stable during follow-up were computed in both MCI samples, resulting in the area under the ROC curve (AUC), which is also known as C-statistics or c-index. The AUC shows the quality of the classification, with 1.0 indicating a perfect discrimination and 0.5 indicating a result obtained by chance only. In order to test whether the resulting AUC derived from *BrainAGE* ROC analysis is statistically greater than the AUCs of cognitive scores, hippocampus volumes, and CSF biomarkers, one-tailed z-tests are performed. Additionally, the McNemar test for paired data was performed in order to statistically test whether predictions of conversion based on baseline *BrainAGE* scores are significantly better than predictions based on cognitive scores, hippocampus volumes, and CSF biomarkers.

Likelihood ratios were computed to determine the likelihood that a *BrainAGE* score or biomarker value above a determined threshold would be expected in pMCI relative to sMCI subjects. These ratios determined whether the use of a clinical biomarker substantially changes the post-test probability that a subject will convert to AD.

Within both MCI samples, univariate Cox regression was used to estimate the hazard rate for conversion to AD, adjusting for age, education years, and gender. The time-to-event variable was time from baseline visit to first visit with AD diagnosis for pMCI subjects. For sMCI subjects, the duration of follow-up was truncated at 3 years. The main predictor was the baseline *BrainAGE* score as a continuous variable initially and in quartiles subsequently. For comparison, Cox regression was also performed with baseline cognitive scores, hippocampus volumes, and CSF biomarkers as main predictors. As checked by log-minus-log-plots of survival, the assumption of proportional hazards was met for all Cox proportional hazard models. Cox regression was performed using SPSS. All other statistical testing was performed using Matlab.

## Results

### Whole MCI Sample

**Figure 2:**
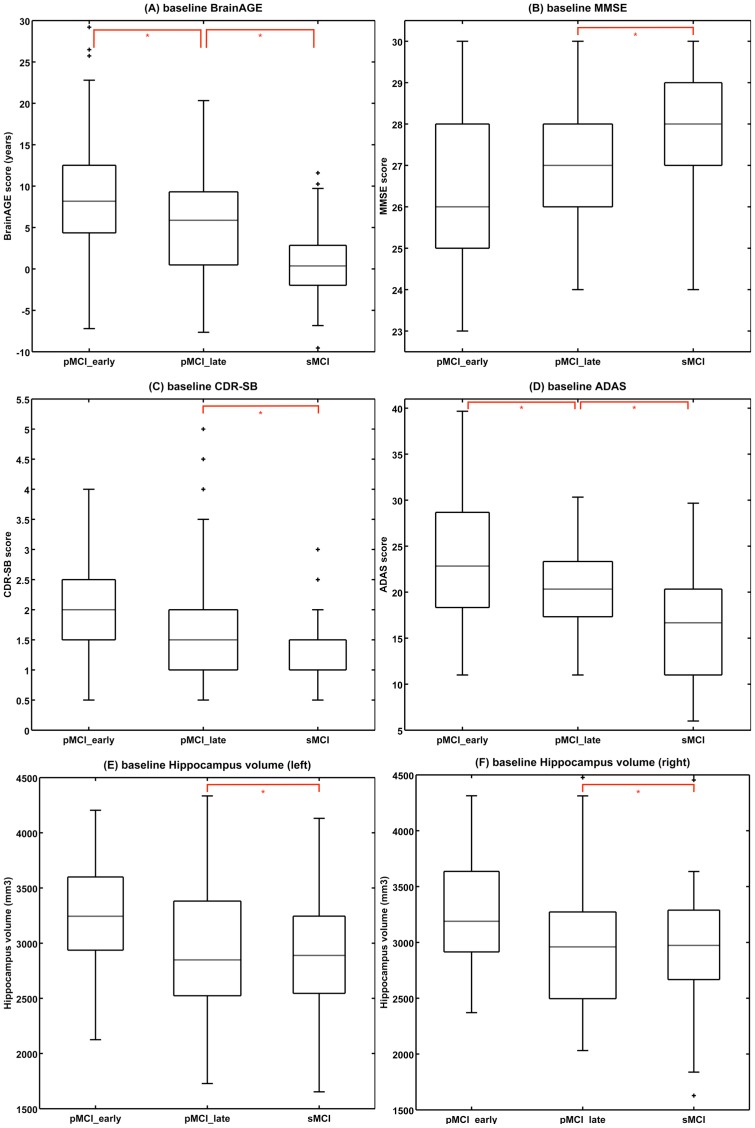
Baseline scores in all MCI groups. Shown are box plots for baseline (A) *BrainAGE* scores (in years), (B) MMSE scores, (C) CDR-SB scores, (D) ADAS scores, (E) left and (F) right hippocampus volumes (in mm^3^) of all diagnostic groups. Post-hoc t-tests resulting in significant differences between diagnostic groups are indicated (p<0.05; red lines). The boxes contain the values between the 25th and 75th percentiles, including the median (dashed line). Lines extending above and below each box symbolize data within 1.5 times the interquartile range (outliers are displayed with a +). Width of the boxes indicates the group size.

The diagnostic groups (i.e. pMCI_early, pMCI_late, sMCI) did not differ in terms age and education years (Table 1). As expected, at baseline examination all cognitive scores as well as the hippocampus volumes differed between groups (Figure 2B–F).

The baseline *BrainAGE* scores significantly differed between the diagnostic groups (F = 26.04; p<0.001), resulting in the following means: pMCI_early = 8.73 years, pMCI_late = 5.62 years, and sMCI = 0.75 years. As mentioned above, positive values indicate a higher estimated than chronological age. Post hoc t-tests showed significant differences (p<0.05) between all three diagnostic groups (Figure 2A).

**Figure 3:**
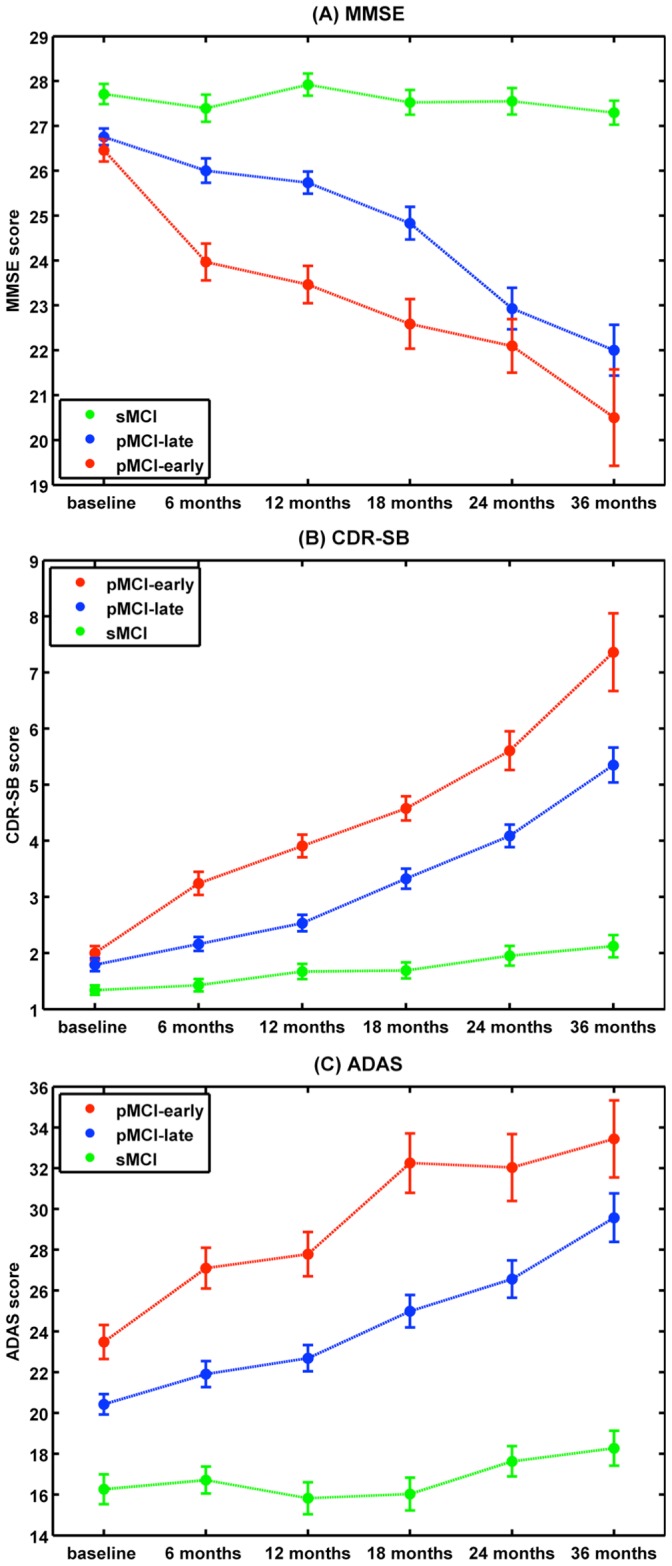
Cognitive scores during follow-up. Mean (A) MMSE, (B) CDR-SB, (C) ADAS scores in pMCI_early, pMCI_late, and sMCI subjects at baseline examination as well as all follow-up assessments. Error bars depict the standard error of the mean (SEM).

As expected, cognitive abilities substantially declined during the follow-up intervals in both pMCI groups but remained stable in those who did not convert to AD (Figure 3A-C). Statistically significant correlations at baseline were only found between *BrainAGE* scores and CDR-SB as well as ADAS, but not for MMSE (Table 3). During follow-up, the correlations between baseline *BrainAGE* scores and clinical disease severity as well as cognitive functioning even increased, denoting a close relationship between pathological brain aging and prospective worsening of cognitive functioning.

**Table 3:** Results for correlation analyses of baseline *BrainAGE* scores with cognitive scores at baseline and follow-up (whole sample).

	baseline	6 months follow-up	12 months follow-up	18 months follow-up	24 months follow-up	36 months follow-up
MMSE	−0.09	−0.17*	−0.25***	−0.24**	−0.39***	−0.41***
CDR-SB	0.20**	0.26***	0.28***	0.32***	0.42***	0.46***
ADAS	0.23**	0.24**	0.35***	0.38***	0.44***	0.48***

*** p<0.001;

** p<0.01;

* p<0.05.

**Figure 4:**
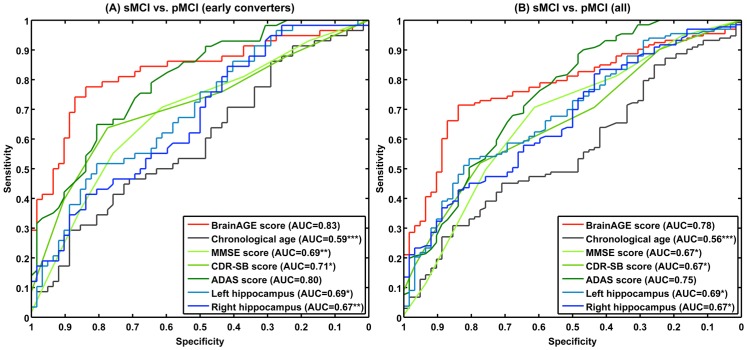
ROC curves of individual subject classification to sMCI or pMCI. ROC curves of individual subject classification to sMCI or pMCI based on baseline *BrainAGE* scores, cognitive scores, and hippocampus volumes for (A) early converters and (B) the whole sample. The areas under the ROC curves (AUCs) of cognitive scores and hippocampus volumes were tested against the AUC of *BrainAGE*: ***p<0.001; **p<0.01; *p<0.05.

Our test sample included 195 subjects diagnosed with MCI at baseline. During 36 months of follow-up, a total of 133 of them developed AD, corresponding to a pre-test probability of 68%. More specifically, 30% of the MCI subjects converted to AD within the first 12 months after baseline examination (mean time to conversion: 312±96 days), whereas 38% of all MCI subjects converted to AD after the first year of follow-up (mean time to conversion: 705±228 days). By varying the threshold applied to the *BrainAGE* score, we constructed ROC curves for a binary discrimination between MCI subjects who remained stable during 3 years follow-up from those who converted to AD. With AUC’s (or c-index) of 0.83 and 0.78, and accuracy rates of 81% and 75% for the discrimination of sMCI vs. pMCI_early (Figure 4A) and all pMCI subjects (Figure 4B), respectively, the baseline *BrainAGE* score proved its encouraging potential to predict conversion to AD in MCI subjects. Furthermore, predicting future conversion to AD based on baseline *BrainAGE* scores was significantly more accurate than predictions based on chronological age, hippocampus volumes, and cognitive scores at baseline (Table 4).

**Table 4:** Results for predicting conversion to AD in MCI subjects with baseline scores (whole sample).

	pMCI_early					pMCI (all)				
	Accuracy [CI]	Sensitivity [CI]	Specificity [CI]	McNemar test		Accuracy [CI]	Sensitivity [CI]	Specificity [CI]	McNemar test	
				Error rate [CI]	*χ^2^*				Error rate [CI]	*χ^2^*
***BrainAGE*** ** score**	**0.81**	**0.78**	**0.84**	**0.19**	***–***	**0.75**	**0.71**	**0.84**	**0.25**	***–***
	**[0.74–0.88]**	**[0.70–0.85]**	**[0.77–0.90]**	**[0.12–0.26]**		**[0.69–0.81]**	**[0.65–0.78]**	**[0.79–0.89]**	**[0.19–0.31]**	
Chronological age	0.41	0.29	0.89	0.59	*28.69*	0.52	0.31	0.85	0.48	*15.87*
	[0.32–0.50]	[0.21–0.37]	[0.83–0.94]	[0.50–0.68]	*[p<0.001]*	[0.45–0.59]	[0.24–0.37]	[0.80–0.90]	[0.41–0.55]	*[p<0.001]*
MMSE score	0.57	0.71	0.61	0.43	*13.07*	0.37	0.71	0.61	0.63	*50.75*
	[0.48–0.66]	[0.63–0.79]	[0.53–0.70]	[0.34–0.52]	*[p<0.001]*	[0.31–0.44]	[0.64–0.77]	[0.54–0.68]	[0.56–0.69]	*[p<0.001]*
CDR-SB score	0.59	0.64	0.77	0.41	*15.87*	0.38	0.52	0.77	0.62	*56.47*
	[0.50–0.68]	[0.55–0.72]	[0.70–0.85]	[0.32–0.50]	*[p<0.001]*	[0.31–0.45]	[0.45–0.59]	[0.72–0.83]	[0.55–0.69]	*[p<0.001]*
ADAS score	0.66	0.65	0.81	0.34	*5.90*	0.48	0.89	0.48	0.52	*31.02*
	[0.57–0.74]	[0.56–0.73]	[0.74–0.88]	[0.26–0.43]	*[p<0.05]*	[0.41–0.55]	[0.84–0.93]	[0.41–0.55]	[0.45–0.59]	*[p<0.001]*
Left hippocampus volume	0.66	0.52	0.81	0.34	*6.42*	0.61	0.53	0.81	0.39	*8.19*
	[0.57–0.74]	[0.43–0.61]	[0.74–0.88]	[0.26–0.43]	*[p<0.05]*	[0.54–0.68]	[0.46–0.60]	[0.75–0.86]	[0.32–0.46]	*[p<0.01]*
Right hippocampus volume	0.61	0.84	0.42	0.39	*9.62*	0.54	0.43	0.84	0.46	*16.00*
	[0.52–0.70]	[0.78–0.91]	[0.33–0.51]	[0.30–0.48]	*[p<0.01]*	[0.47–0.61]	[0.36–0.50]	[0.79–0.89]	[0.39–0.53]	*[p<0.001]*

**Figure 5:**
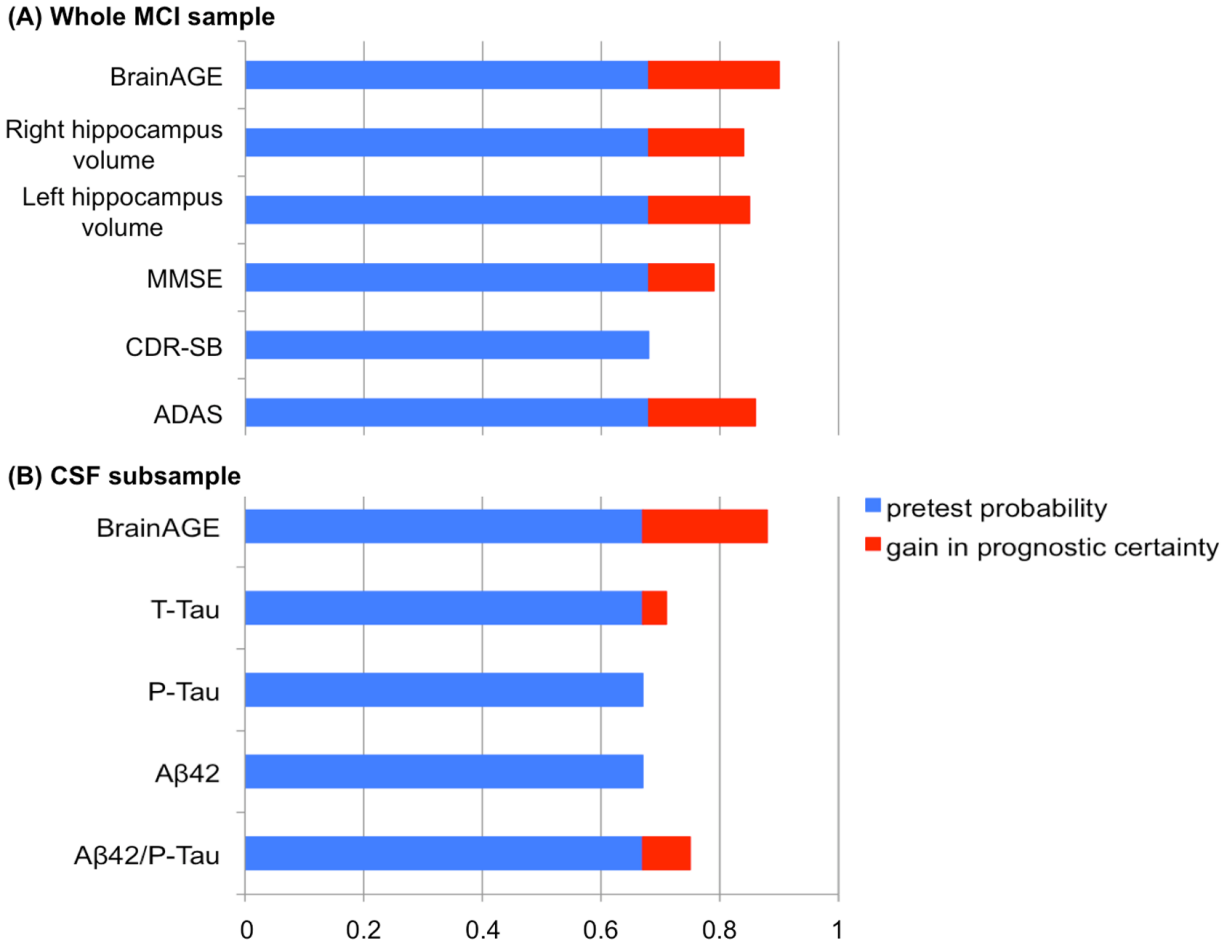
Pre-test and post-test probability for predicting conversion to AD. Pre-test probability (blue) and post-test probability (blue+red), indicating the gain in prognostic certainty (red) for predicting conversion to AD within 36 months, based on (A) baseline *BrainAGE* scores, hippocampus volume, and cognitive measures within the whole MCI sample, as well as (B) baseline *BrainAGE* scores and CSF biomarkers in the CSF subsample.

For the whole MCI sample the post-test probability was increased to 90% when using baseline *BrainAGE* scores to predict conversion to AD within 36 months of follow-up (Figure 5A). This gain in certainty by 22% was highest for the baseline *BrainAGE* score as compared to baseline hippocampus volumes (right hippocampus: 16%; left hippocampus: 17%) or cognitive scores (MMSE: 11%; CDR-SB: 0%; ADAS: 18%).

**Figure 6:**
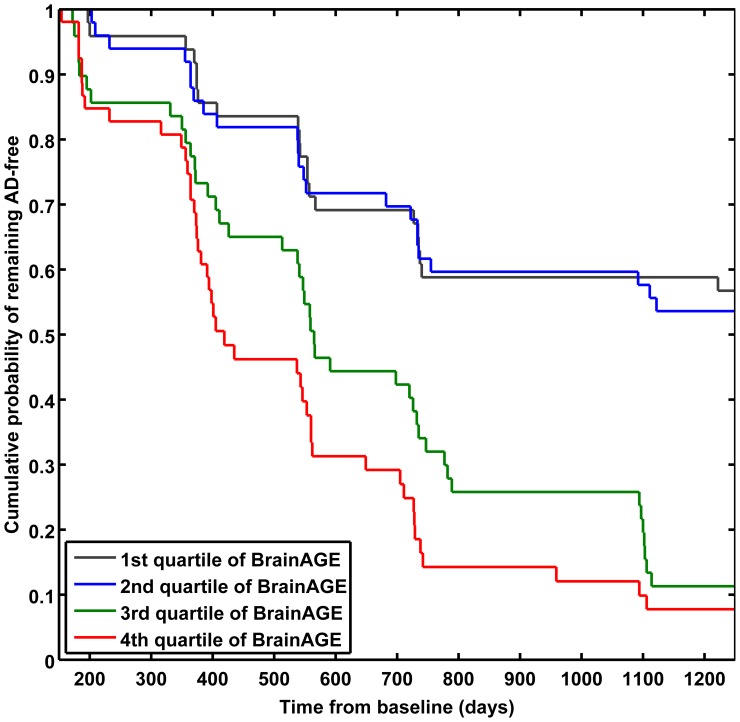
Cumulative probability of remaining AD-free in the quartiles of baseline ***BrainAGE*** **score.** Kaplan-Meier survival curves based on Cox regression comparing cumulative AD incidence in subjects with MCI at baseline by *BrainAGE* score quartiles (p for trend <0.001). Duration of follow-up is truncated at 1250 days.

Cox regression analysis showed an association of higher *BrainAGE* scores with a higher risk of developing AD (χ^2^ = 58.86, p<0.001; Table 5). Each additional year in the *BrainAGE* score was associated with a 10% greater risk of developing AD (hazard rate: 1.10, p<0.001; Table 5). Compared with subjects in the lowest *BrainAGE* quartile (−9.55 – −0.12 years), subjects in the 2^nd^ quartile (−0.12–4.45 years) had about the same risk of developing AD (hazard ratio [HR]: 1.13; CI: 0.62–2.06; p = 0.68), those in the 3^rd^ quartile (4.46–9.26 years) had a three times greater risk (HR: 3.12; CI: 1.80–5.40; p<0.001), and those in the 4^th^ quartile (9.26–29.20 years) had a four times greater risk (HR: 4.66; CI: 2.61–8.29; p<0.001) of developing AD (Figure 6). Thus, MCI subjects showing abnormal atrophy patterns as marked by higher *BrainAGE* scores had a significantly increased risk and a cumulative probability of 88% in the 3^rd^ quartile and 92% in the 4^th^ quartile for conversion to AD. Furthermore, when performing Cox regression with all other baseline scores, *BrainAGE* again showed the best results (Table 5; Figure S1).

**Table 5:** Model statistics of Cox regression for all baseline scores (adjusted for age, gender, and education).

	Continuous predictors					Categorical predictors (median split)				
	Overall model		Continuous values			Overall model		Values below vs. above median		
	χ^2^	p	Hazard rate[CI]	Wald statistics	p	χ^2^	p	Hazard ratio [CI]	Wald statistics	p
***BrainAGE*** ** score (+)**	**58.86**	***	**1.10**	**45.05**	***	**52.23**	***	**3.41**	**37.03**	***
			**[1.07–1.13]**					**[2.30–5.07]**		
MMSE score (-)	28.99	***	0.81	16.01	***	25.04	***	2.02	12.55	***
			[0.73–0.90]					[1.37–2.99]		
CDR-SB score (+)	30.46	***	1.15	19.41	***	26.74	***	1.97	13.89	***
			[1.26–1.82]					[1.38–2.82]		
ADAS score (+)	56.02	***	1.11	40.48	***	29.78	***	2.12	16.84	***
			[1.07–1.14]					[1.48–3.03]		
Left hippocampus volume (-)	34.54	***	1.00	21.82	***	23.84	***	1.91	11.34	**
			[1.00–1.00]					[1.31–2.78]		
Right hippocampus volume (-)	31.65	***	1.00	18.90	***	18.56	**	1.59	6.32	*
			[1–00–1.00]					[1.11–2.28]		

(+) = higher values mean higher risk for AD; (-) = lower values mean higher risk for AD.

*** p<0.001;

** p<0.01;

* p<0.05; n.s. = not significant.

**Bold** type = best performance of all markers.

### MCI Subsample with CSF Data

**Figure 7:**
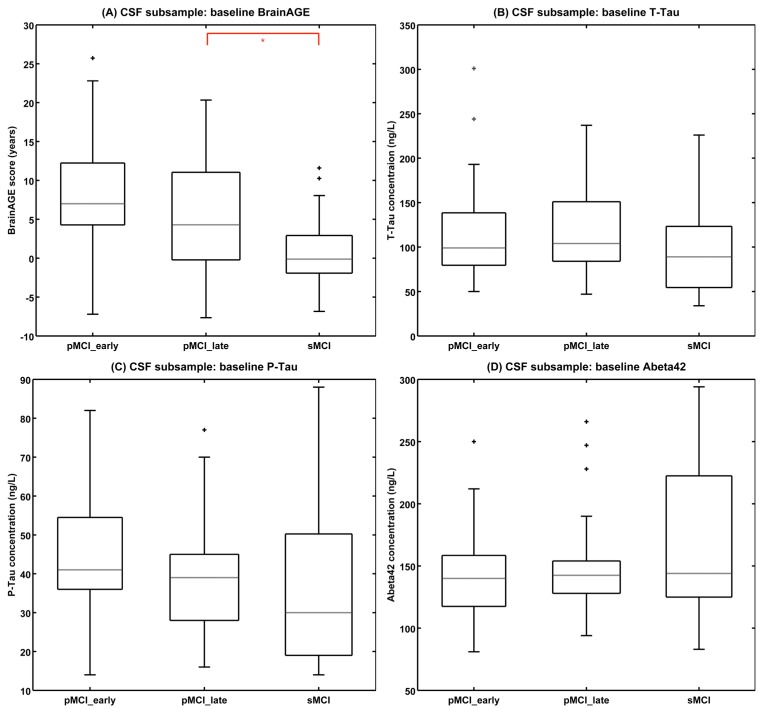
Baseline *BrainAGE* scores and baseline CSF biomarker concentrations in the MCI-subsample. Shown are box plots for (A) *BrainAGE* scores, (B) T-Tau, (C) P-Tau, and (D) Aβ_42_ concentration at baseline of all diagnostic groups in the subsample that also provides CSF data. The boxes contain the values between the 25th and 75th percentiles, including the median (grey line). Lines extending above and below each box symbolize data within 1.5 times the interquartile range (outliers are displayed with a +). Width of the boxes indicates the group size. Post-hoc t-tests resulted in significant differences between diagnostic groups only for baseline *BrainAGE* scores (p<0.05; red lines).

When comparing *BrainAGE* to state-of-the-art CSF biomarkers within this multi-center study, only the baseline *BrainAGE* scores significantly differed between the diagnostic groups in the CSF subsample (sMCI^CSF^: 0.71 years; pMCI^CSF^_late: 5.04; pMCI^CSF^_early: 8.20; F = 10.82, p<0.001; Figure 7), but none of the baseline CSF biomarker levels (Table 1). *BrainAGE* scores in the CSF subsample did not differ between from those in the whole MCI sample (F = 0.15, p = 0.86).

**Figure 8:**
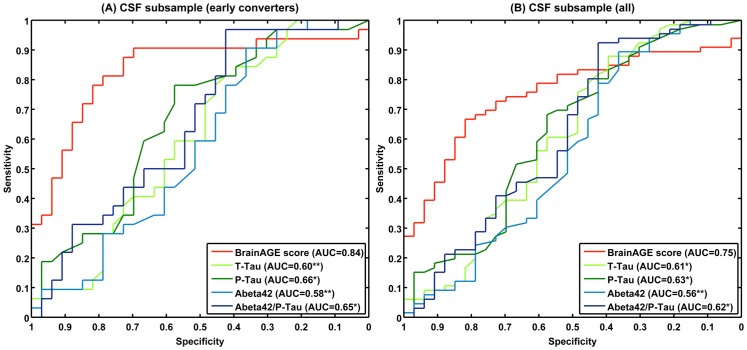
ROC curves of individual subject classification to sMCI or pMCI in the CSF subsample. ROC curves of individual subject classification to sMCI^CSF^ or pMCI^CSF^ based on baseline *BrainAGE* scores and CSF biomarkers for (A) early converters and (B) the whole CSF subsample. The areas under the ROC curves (AUCs) of the CSF biomarkers were tested against the AUC of *BrainAGE*: **p<0.01; *p<0.05.

ROC analyses with baseline *BrainAGE* scores resulted in AUC’s (or c-index) of 0.84 and 0.75, and accuracy rates accuracy rates of 80% and 72% for the discrimination of sMCI^CSF^ vs. pMCI^CSF^ in early converters (Figure 8A) and in the whole CSF subsample (Figure 8B), respectively. Thus, baseline *BrainAGE* scores showed significantly better predictions than baseline T-Tau, P-Tau, and Aβ_42_ levels (Table 6).

**Table 6:** Results in the CSF subsample for predicting conversion to AD in MCI subjects with baseline scores.

	pMCI^CSF^_early					pMCI^CSF^ (all)				
	Accuracy [CI]	Sensitivity [CI]	Specificity [CI]	McNemar test		Accuracy [CI]	Sensitivity [CI]	Specificity [CI]	McNemar test	
				Error rate [CI]	*χ^2^*				Error rate [CI]	*χ^2^*
***BrainAGE*** ** score**	**0.80**	**0.91**	**0.70**	**0.20**	***–***	**0.72**	**0.67**	**0.82**	**0.28**	***–***
	**[0.70–0.90]**	**[0.83–0.98]**	**[0.58–0.81]**	**[0.10–0.30]**		**[0.63–0.81]**	**[0.57–0.76]**	**[0.74–0.89]**	**[0.19–0.37]**	
T-Tau	0.60	0.84	0.39	0.40	*4.80*	0.58	0.88	0.39	0.42	*3.93*
	[0.48–0.72]	[0.76–0.93]	[0.27–0.51]	[0.28–0.52]	*[p<0.05]*	[0.48–0.76]	[0.81–0.94]	[0.30–0.49]	[0.33–0.52]	*[p<0.05]*
P-Tau	0.57	0.78	0.58	0.43	*7.54*	0.43	0.68	0.58	0.57	*16.20*
	[0.45–0.69]	[0.68–0.88]	[0.46–0.70]	[0.31–0.55]	*[p<0.01]*	[0.34–0.53]	[0.59–0.77]	[0.48–0.67]	[0.47–0.66]	*[p<0.001]*
Aβ_42_	0.57	0.91	0.36	0.43	*7.26*	0.49	0.89	0.36	0.51	*7.08*
	[0.45–0.69]	[0.83–0.98]	[0.25–0.48]	[0.31–0.55]	*[p<0.01]*	[0.40–0.59]	[0.83–0.95]	[0.27–0.46]	[0.41–0.60]	*[p<0.01]*
Aβ_42_/P-Tau	0.69	0.97	0.42	0.31	*1.69*	0.73	0.92	0.42	0.27	*0.03*
	[0.58–0.80]	[0.93–1.00]	[0.30–0.54]	[0.20–0.42]	*[n.s.]*	[0.64–0.81]	[0.87–0.98]	[0.33–0.52]	[0.18–0.36]	*[n.s.]*

n.s. = not significant.

Furthermore, when looking at the post-test probability in the CSF subsample, the pre-test probability of 67% for converting to AD within three years was increased by 21% using baseline *BrainAGE* scores (Figure 5B), but only slightly by using CSF biomarkers (T-Tau: 4%, P-Tau: 0%, Aβ_42_∶0%, Aβ_42_/P-Tau: 8%).

Also in the CSF subsample, Cox regression analysis showed a significant association of higher *BrainAGE* scores with a higher risk of developing AD (χ^2^ = 22.11, p<0.001; Table 5). In contrast, Cox regression with CSF biomarkers did not yield significant results for any of them (Table 7, Figure S2).

**Table 7:** Model statistics of Cox regression for CSF-biomarker baseline levels in the CSF subsample (adjusted for age, gender, and education).

	Continuous predictors					Categorical predictors (median split)				
	Overall model		Continuous values			Overall model		Values below vs. above median		
	χ^2^	p	Hazard rate [CI]	Wald statistics	p	χ^2^	p	Hazard ratio [CI]	Wald statistics	p
***BrainAGE*** ** score (+)**	**22.11**	***	**1.08**	**14.86**	***	**24.33**	***	**3.41**	**16.75**	***
			**[1.04–1.12]**					**[1.89–6.14]**		
T-Tau (+)	6.90	n.s.	1.00	0.17	n.s.	6.92	n.s.	1.11	0.15	n.s.
			[1.00–1.01]					[0.67–1.84]		
P-Tau (+)	9.54	*	1.01	2.78	n.s.	7.86	n.s.	1.29	0.96	n.s.
			[1.00–1.03]					[0.77–2.14]		
Aβ_42_ (***–***)	10.33	*	0.99	3.54	n.s.	6.73	n.s.	0.98	0.00	n.s.
			[0.99–1.00]					[0.59–1.63]		
Aβ_42_/P-Tau (***–***)	12.90	*	0.91	5.63	*	6.83	n.s.	1.08	0.10	n.s.
			[0.84–0.98]					[0.66–1.78]		

(+) = higher values mean higher risk for AD; (***–***) = lower values mean higher risk for AD;

*** p<0.001;

* p<0.05; n.s. = not significant.

**Bold** type = best performance of all markers.

## Discussion

The scope of this study was the implementation of a novel MRI-based biomarker based on the recently presented *BrainAGE* framework [25] to predict prospective cognitive decline and conversion to AD on an individual subject level. Using structural MRI data, our fully automated age estimation model aggregates the complex, multidimensional aging patterns across the whole brain to one single value (i.e. the *BrainAGE* score) and finally identifies pathological brain aging in MCI subjects who finally converted to AD within three years of follow-up, with increasing *BrainAGE* scores at baseline indicating an increased risk of developing AD.

This method already showed the advantage of accurately and reliably estimating the age of the brain with minimal preprocessing and parameter optimization [25], [26], using a single anatomical scan. Regarding the relevance within the clinical context, higher *BrainAGE* scores were recently demonstrated to be closely related to measures of clinical disease severity in AD patients, as well as prospective worsening of cognitive functioning in MCI subjects who converted to AD within three years [27]. Furthermore, already possessing higher *BrainAGE* scores at baseline, brain atrophy was shown to even accelerate during follow-up, with the speed of one additional year per follow-up year in pMCI subjects and 1.5 additional years per follow-up year in AD patients. Considering unequal follow-up durations in the pMCI and AD groups, this finally accumulated to mean *BrainAGE* scores of about 9 years at the last scan in both groups. Compared to that, sMCI and healthy control subjects did not show any irregularity in brain atrophy at baseline and follow-up [27].

In the study presented here, the *BrainAGE* approach was implemented to predict subsequent conversion to AD on a single subject level based on structural MRI at baseline. Focusing on subjects with mild memory impairment but preserved activities of daily life, we found accuracy rates of up to 81% for prediction of progression to AD. Even more interestingly, a high *BrainAGE* score increased the prognostic certainty of a subsequent conversion to AD from 68% in our clinically defined MCI sample to 90%. This gain in certainty may provide solid diagnostic grounds for early intervention strategies aimed at delaying or preventing the onset of full-scale AD in subjects at highest risk for the disease. Furthermore, our *BrainAGE* framework was more precise in predicting conversion of MCI to AD when compared to chronological age, cognitive scores, hippocampus volume, or state-of-the-art CSF biomarkers.

Cognitive decline was recently found to progressively accelerate years before being diagnosed as AD [42], and to be correlated with the atrophy rates in specified brain regions [43]. In addition, some studies focusing on regression methods to identify pathological brain structures specific for AD reported moderate performance measures when predicting one-year decline of cognitive functions in MCI [44]–[46]. Although not specifically trained to predict changes in cognitive scales, the *BrainAGE* scores estimated at baseline showed moderate correlations with measures of clinical disease severity and cognitive functioning up to three years in advance. These results as well as our recent results from a longitudinal *BrainAGE* study [26] support the suggested relationship between progressive acceleration in brain atrophy and worsening of cognitive functioning in progressive MCI.

Using high-dimensional pattern recognition with imaging data was recently suggested to provide a viable biomarker to detect subtle, but predictive, imaging phenotypes that precede cognitive decline while there is still opportunity for preventive or therapeutic interventions [47]. Current classification approaches attempt to identify disease-specific patterns that allow a separation of subjects with MCI or AD from healthy samples. Whilst most approaches are able to accurately differentiate between healthy controls and AD patients [48]–[50], it is the conversion from MCI to AD that is of greater clinical interest and clinical consequence. Attempting this issue, most approaches showed a substantial drop in accuracy when predicting MCI-to-AD conversion on an individual level, especially when relying on baseline data only [23], [51]–[55]. Nevertheless, individuals showing the first subtle signs of abnormal atrophy will benefit most from an early therapy, provided to reliably identify those individuals at risk of progressing to AD in future. For example, a recent study based on cortical thickness reported the accurate detection of 81% of those MCI subjects who were to be clinically diagnosed as AD patients 24 months later [52]. But this was only true when looking at those MCI subjects who were converting to AD, while ignoring those MCI subjects who did not convert. Consequently, the overall accuracy of sMCI vs. pMCI classification ranged from 48% at 6 months to 73% at 24 months. Furthermore, although a very recent study reported that combining MRI and CSF measures in a multivariate model resulted in better accuracy for predicting future conversion from MCI to AD, than using either MRI or CSF separately [56], the overall prediction accuracies for converters and non-converters ranged only from 58.6% to 66.4% at different time points. With sensitivity of 67% and specificity of 69%, another recent study [57] also achieved the most stable and reliable classification results when combining all available structural MRI features (i.e. hippocampus volume, tensor-based morphometry, cortical thickness). Thus, with accuracy rates up to 81% in predicting conversion to AD within the whole MCI sample up to three years in advance, *BrainAGE* is comparable or even outperforms recent classification studies that predicted decline of cognitive scores in MCI subjects or short-term conversion to AD (e.g., [51], [52]–[55], [58]).

Besides and in contrast to CSF biomarkers, MRI is non-invasive and can be performed more rapidly than a detailed neuropsychological testing. Furthermore, brain imaging is part of the diagnostic work-up [49], with MRI becoming the imaging modality of choice in many centers. Additionally, MRI was shown to retain the closest relationship with memory loss as well as worsening of clinical functions [2]. Consequently, current models of the dynamics of well established biomarkers of the Alzheimer’s pathological cascade suggest a crucial role for structural MRI in predicting future cognitive decline and conversion to AD [2], [24], [47]. Even though hippocampus volume has been shown to represent an independent risk factor for AD and robustly predicting conversion to AD in MCI subjects, the *BrainAGE* approach outperformed prediction utilizing baseline hippocampus volumes in the present study as well as in recently published classification studies [59]–[61].

One limitation of our approach might be that white matter lesions that occur primarily due to cerebro-vascular diseases are not detected in the segmentation approach. Those lesions are segmented as gray matter and might therefore influence the relevance vector regression. However, because such lesions only occur in a limited number of subjects it is very unlikely that they contribute to the relevance vectors because of their high local variance. In future, the segmentation should be extended by methods that allow an automated detection of white matter lesions even without any additional FLAIR sequence [62].

As stated before, the *BrainAGE* method builds on the assumption of AD being preceded by an acceleration in brain atrophy that resembles advanced aging (e.g., [3], [4]–[8]), although there are other studies rejecting that assumption (e.g., [63], [64]). However, the acceleration of spatiotemporal brain atrophy might only be seen in subjects in a preclinical stage, while in AD patients additional disease-specific pathological changes are occurring. Further, subjects with a high *BrainAGE* score but no AD-specific clinical profile may suffer from other neurodegenerative diseases. This issue should be explored by applying our framework to other neurodegenerative diseases. Furthermore, cognitive reserve, genetic status, education level, socioeconomic status, lifestyle, or vitamin supply may protect subjects from pathological brain aging or accelerated cognitive decline despite high *BrainAGE* scores [52], [65]–[68]. Thus, in future research we aim to disentangle age- and unrelated disease-based processes of brain tissue loss in AD. Additionally, we will elucidate the effects of the genetic status (e.g. Apolipoprotein E (APOE)) on the longitudinal changes in *BrainAGE* as well as on prediction of AD conversion, since especially the APOE ε4 allele is associated with modification of cognitive functioning [69]–[71] and GM reduction in AD patients [72] as well as healthy subjects [73].

In conclusion, *BrainAGE* has shown promising results on an individual level, contributing to an early indication of pathological brain aging in advance of severe clinical symptoms, or even predicting future cognitive decline. Compared to a wide range of existing classification approaches that require disease-specific data for training, the *BrainAGE* framework uses an independent database of healthy, non-demented subjects to model the normal brain-aging pattern and consequently recognizing subtle deviations from age-related brain atrophy in new test samples. As the *BrainAGE* approach utilizes only a single T1-weighted image per subject and already has proven to work fast and fully automated with multi-centre data, it can be easily implemented in clinical routine to encourage the identification of subtly abnormal atrophy patterns.

## Supporting Information

Figure S1 Cumulative probability of remaining AD-free in the whole MCI sample. Kaplan-Meier survival curves based on Cox regression comparing cumulative AD incidence in subjects with MCI at baseline by all baseline scores split at median. Duration of follow-up is truncated at 1250 days.
(TIFF)

Figure S2 Cumulative probability of remaining AD-free in the CSF subsample. Kaplan-Meier survival curves based on Cox regression comparing cumulative AD incidence in subjects with MCI at baseline by all CSF biomarker baseline levels split at median. Duration of follow-up is truncated at 1250 days.
(TIFF)

Table S1 Subject IDs from the ADNI database of the MCI test samples used in this study (subjects of the CSF subsample are indicated).
(DOCX)
