# Supplementary figures and images for: BrainAGE in Mild Cognitive Impaired Patients: Predicting the Conversion to Alzheimer’s Disease
Source: PLoS One. 2013 Jun 27;8(6):e67346. doi: 10.1371/journal.pone.0067346 (PMC3695013; doi:10.1371/journal.pone.0067346)

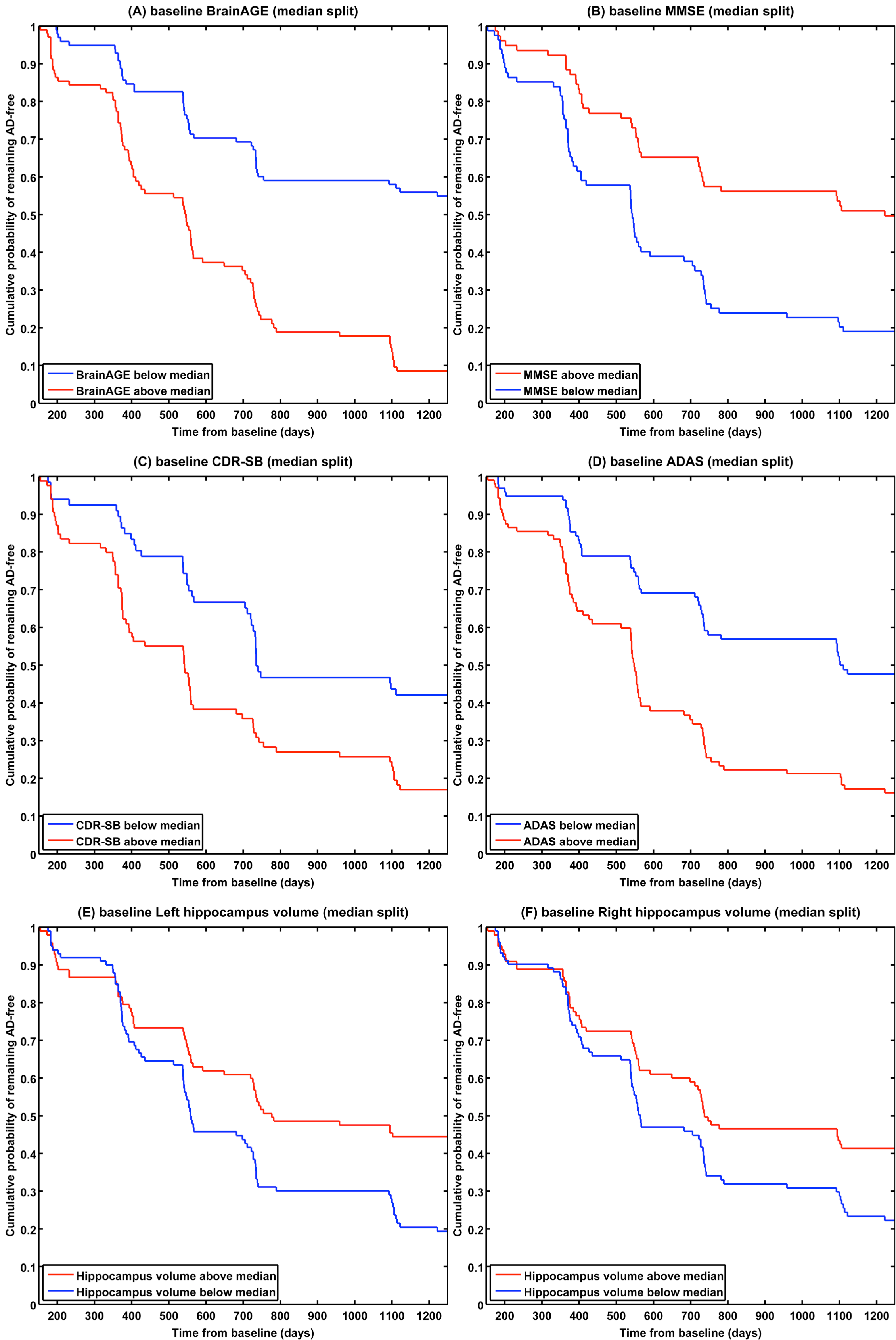

Supplement: Figure S1 — Cumulative probability of remaining AD-free in the whole MCI sample. Kaplan-Meier survival curves based on Cox regression comparing cumulative AD incidence in subjects with MCI at baseline by all baseline scores split at median. Duration of follow-up is truncated at 1250 days. (TIFF) [file pone.0067346.s001.tiff]

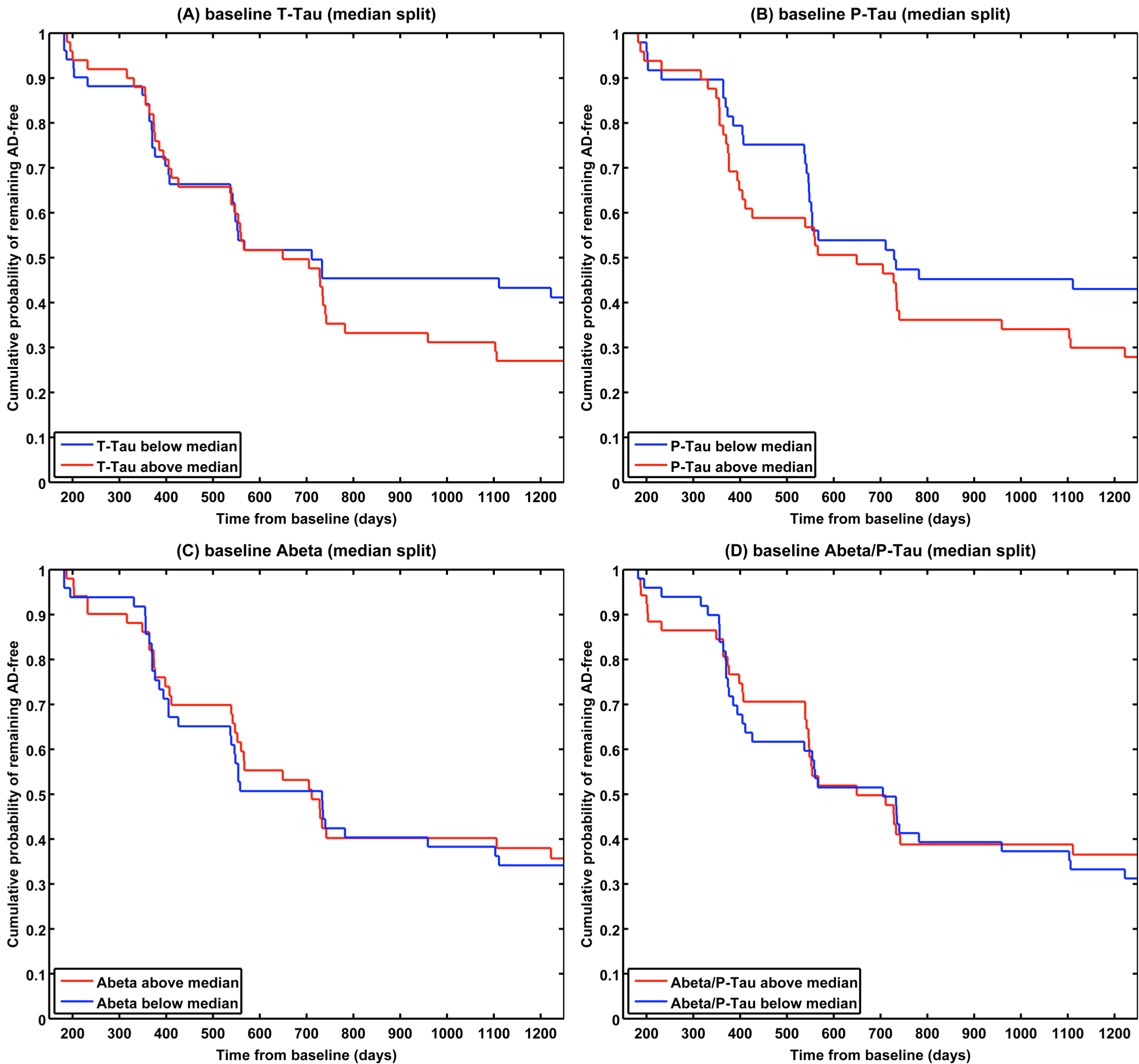

Supplement: Figure S2 — Cumulative probability of remaining AD-free in the CSF subsample. Kaplan-Meier survival curves based on Cox regression comparing cumulative AD incidence in subjects with MCI at baseline by all CSF biomarker baseline levels split at median. Duration of follow-up is truncated at 1250 days. (TIFF) [file pone.0067346.s002.tiff]
