# Supplementary material for: BrainAGE in Mild Cognitive Impaired Patients: Predicting the Conversion to Alzheimer’s Disease
Source: PLoS One. 2013 Jun 27;8(6):e67346. doi: 10.1371/journal.pone.0067346 (PMC3695013; doi:10.1371/journal.pone.0067346)
Supplement: Table S1 — Subject IDs from the ADNI database of the MCI test samples used in this study (subjects of the CSF subsample are indicated). (DOCX) [file pone.0067346.s003.docx]

| **pMCI_early** | | **pMCI_late** | | **sMCI** | |
| --- | --- | --- | --- | --- | --- |
| 203 ^(CSF)^ | 472 | 208 | 353 ^(CSF)^ | 202 | 393 |
| 211 ^(CSF)^ | 474 ^(CSF)^ | 215 | 354 | 205 | 415 |
| 231 | 484 ^(CSF)^ | 219 | 358 ^(CSF)^ | 206 ^(CSF)^ | 428 |
| 237 | 498 | 220 ^(CSF)^ | 359 ^(CSF)^ | 207 ^(CSF)^ | 435 ^(CSF)^ |
| 240 | 499 ^(CSF)^ | 221 ^(CSF)^ | 368 ^(CSF)^ | 210 | 438 |
| 247 ^(CSF)^ | 505 | 222 ^(CSF)^ | 372 | 212 | 440 ^(CSF)^ |
| 250 ^(CSF)^ | 506 | 223 | 374 | 213 ^(CSF)^ | 449 ^(CSF)^ |
| 254 | 518 ^(CSF)^ | 224 | 375 | 214 | 459 ^(CSF)^ |
| 261 ^(CSF)^ | 525 | 225 ^(CSF)^ | 376 | 228 | 468 |
| 299 | 560 | 226 ^(CSF)^ | 381 | 229 ^(CSF)^ | 471 ^(CSF)^ |
| 300 ^(CSF)^ | 562 ^(CSF)^ | 241 ^(CSF)^ | 383 ^(CSF)^ | 242 | 475 ^(CSF)^ |
| 309 ^(CSF)^ | 567 ^(CSF)^ | 252 ^(CSF)^ | 391 | 259 | 479 ^(CSF)^ |
| 310 ^(CSF)^ | 570 | 260 | 395 ^(CSF)^ | 274 | 490 ^(CSF)^ |
| 314 ^(CSF)^ | 573 | 269 | 409 | 277 | 494 |
| 319 ^(CSF)^ | 580 | 272 | 420 ^(CSF)^ | 287 ^(CSF)^ | 495 |
| 329 ^(CSF)^ | 593 | 273 ^(CSF)^ | 423 | 288 ^(CSF)^ | 496 ^(CSF)^ |
| 344 | 594 | 278 | 430 | 294 ^(CSF)^ | 511 |
| 348 | 598 | 279 | 434 | 295 ^(CSF)^ | 514 ^(CSF)^ |
| 356 ^(CSF)^ | 603 | 280 | 450 | 305 ^(CSF)^ | 520 ^(CSF)^ |
| 357 ^(CSF)^ | 604 | 283 ^(CSF)^ | 470 | 312 ^(CSF)^ | 521 |
| 360 |  | 292 ^(CSF)^ | 497 ^(CSF)^ | 317 | 522 ^(CSF)^ |
| 366 ^(CSF)^ |  | 302 ^(CSF)^ | 501 | 320 ^(CSF)^ | 540 ^(CSF)^ |
| 398 |  | 303 | 502 | 321 | 564 |
| 401 ^(CSF)^ |  | 304 ^(CSF)^ | 507 | 325 ^(CSF)^ | 565 ^(CSF)^ |
| 406 |  | 306 ^(CSF)^ | 508 ^(CSF)^ | 328 ^(CSF)^ |  |
| 411 ^(CSF)^ |  | 307 ^(CSF)^ | 510 | 335 |  |
| 416 ^(CSF)^ |  | 311 | 512 | 339 |  |
| 421 ^(CSF)^ |  | 315 ^(CSF)^ | 513 ^(CSF)^ | 343 |  |
| 424 ^(CSF)^ |  | 318 | 538 | 346 |  |
| 432 ^(CSF)^ |  | 323 | 541 ^(CSF)^ | 347 |  |
| 433 ^(CSF)^ |  | 326 ^(CSF)^ | 551 ^(CSF)^ | 351 ^(CSF)^ |  |
| 436 ^(CSF)^ |  | 332 | 558 | 363 |  |
| 439 ^(CSF)^ |  | 334 ^(CSF)^ | 563 | 365 ^(CSF)^ |  |
| 442 |  | 337 ^(CSF)^ | 574 | 367 ^(CSF)^ |  |
| 457 |  | 342 ^(CSF)^ | 575 | 370 ^(CSF)^ |  |
| 461 |  | 349 | 592 ^(CSF)^ | 373 |  |
| 466 ^(CSF)^ |  | 350 | 602 ^(CSF)^ | 377 ^(CSF)^ |  |
| 469 ^(CSF)^ |  | 352 ^(CSF)^ |  | 380 ^(CSF)^ |  |
